# The Correlation between miRNA and Lymph Node Metastasis in Gastric Cancer

**DOI:** 10.1155/2015/543163

**Published:** 2015-01-22

**Authors:** Kuo-Hung Huang, Yuan-Tzu Lan, Wen-Liang Fang, Jen-Hao Chen, Su-Shun Lo, Anna Fen-Yau Li, Shih-Hwa Chiou, Chew-Wun Wu, Yi-Ming Shyr

**Affiliations:** ^1^Division of General Surgery, Department of Surgery, Taipei Veterans General Hospital, No. 201, Sec. 2, Shipai Road, Beitou District, Taipei City 11217, Taiwan; ^2^School of Medicine, National Yang-Ming University, Taipei City 11221, Taiwan; ^3^Institute of Clinical Medicine, School of Medicine, National Yang-Ming University, Taipei City 11221, Taiwan; ^4^Division of Colon & Rectal Surgery, Department of Surgery, Taipei Veterans General Hospital, Taipei City 11217, Taiwan; ^5^En Chu Kong Hospital, New Taipei City 237, Taiwan; ^6^National Yang-Ming University Hospital, Yilan City 260, Taiwan; ^7^Department of Pathology, Taipei Veterans General Hospital, Taipei City 11217, Taiwan; ^8^Department of Medical Research and Education, Taipei Veterans General Hospital, Taipei City 11217, Taiwan; ^9^Institute of Pharmacology, National Yang-Ming University, Taipei City 11221, Taiwan

## Abstract

Lymph node metastasis (LNM) in gastric cancer is associated with higher rate of cancer recurrence and poor prognosis. As a result, a reliable biomarker for the prediction of LNM is important and would be valuable in the clinical practice. MiRNA microarray revealed that ten miRNAs were expressed significantly different among patients with or without LNM. A total of 46 gastric cancer patients were enrolled and divided into two groups (23 in each group) according to the presence or absence of LNM. RT-PCR of these 10 miRNAs was investigated and compared between the two groups. MiR-1207-5p was significantly upregulated in gastric cancer patients without LNM compared with those with LNM. Patients with upregulated miR-1207-5p had less scirrhous stromal reaction, less lymphovascular invasion, and earlier pathological T category, N category, and TNM stage, compared with those with downregulated or unchanged miR-1207-5p. Multivariate analysis showed that stromal reaction type, lymphovascular invasion, pathological T category and TNM stage, and expression of miR-1207-5p were independent risk factors of LNM. MiR-1207-5p could serve as a useful biomarker in the prediction of LNM in gastric cancer.

## 1. Introduction

Although the incidence of gastric cancer worldwide has been declining, it remains the fourth most common cancer and the second most common cause of cancer death worldwide [1]. About 90% of recurrence after curative surgery of gastric cancer happened within 3 years and is usually associated with poor outcome.

The risk factors of recurrence after gastric cancer surgery include lymph node metastasis (LNM), depth of cancer invasion, stromal reaction, and gross appearance. However, the discovery of new biomarkers for cancer prognostic prediction is vigorous day by day, including gastric cancer.

LNM is one of the most important risk factors of gastric cancer recurrence after curative surgery. Lymph node dissection has been proved to improve survival of gastric cancer [2]. Our previous study [3] showed that the 5 yr survival rates of each pathological N category were as follows: N0 (83.5%), N1 (57.8%), N2 (27.4%), and N3 (11.4%), *P* < 0.001. As a result, pathological N category is one of the independent prognostic factors of gastric cancer.

It has been demonstrated that the plasma concentrations of various miRNAs, such as miR-17-5p, miR-21, miR-106a, and miR-106b, are higher whereas let-7a is lower in gastric cancer patients. The value of the area under the receiver-operating characteristic curve can be achieved as high as 0.879 for the miR-106a/let-7a ratio assay [4]. High levels of miR-17 and miR-106a in peripheral blood of gastric cancer patients have also been confirmed in another study in which the value of the area under the receiver-operating characteristic curve for combined miR-17/miR-106a assay was 0.741 [5].

These findings suggest that miRNAs are useful biomarkers for early diagnosis of gastric cancer. It is expected that the incorporation of miRNA into current panels of biomarkers may enhance the sensitivity and specificity of noninvasive diagnostic tests for gastric cancer.

MiRNAs have recently been used to predict the outcome of patients with gastric cancer. For example, a seven-miRNA signature (miR-10b, miR-21, miR-223, miR-338, let-7a, miR-30a-5p, and miR-126) is closely associated with relapse-free and overall survival among patients with gastric cancer [6]. High expression levels of miR-20b or miR-150 [7], or downregulation of miR-451 [8] or miR-218 [9], are also associated with poor survival, whereas there is a correlation between miR-27a and lymph node metastasis [7]. In addition, Ueda et al. [10] recently reported that miR-125b, miR-199a, and miR-100 represent a progression-related signature, whereas low expression of let-7g and high expression of miR-214 are associated with shorter overall survival independent of depth of invasion, LNM, and stage. These prognostic miRNAs could be applicable to future decisions concerning treatment.

In esophageal squamous cell carcinoma, upregulation of miR-92a was significantly correlated with the status of LNM and TNM stage [11]. Tchernitsa et al. [12] reported six miRNAs separating node-positive from node-negative gastric cancers, that is, miR-103, miR-21, miR-145, miR-106b, miR-146a, and miR-148a. Up to now, there have been only few reports investigating the relationship between miRNA and LNM in gastric cancer.

In this study, we will investigate the difference of miRNA profiling between node-negative and node-positive gastric cancer tissue. MiRNAs which are significantly upregulated or downregulated with regard to the clinicopathological characteristics will be analyzed. We hope to discover specific miRNAs which are expressed diversely between different pathological T, N, or TNM stage. We hope to discover new miRNAs as useful biomarker for predicting LNM. These selected miRNAs might also remind the surgeon of the possibility of LNM in high risk patients and the need of careful lymph node dissection for these patients during surgery.

## 2. Material and Methods

### 2.1. Patients and Tumor Tissues

Primary gastric cancer tissues and their corresponding normal mucosa were obtained from patients at Taipei Veterans General hospital. Clinical data were prospectively collected. The institutional review board at the Taipei Veterans General Hospital approved this study, and written informed consent was obtained from all of the patients.

The neoplasms were dissected meticulously and the tumor tissues and corresponding normal mucosa were collected. Tissue fragments were frozen immediately in liquid nitrogen and stored at −70°C. Sections of malignant and collateral tissues were reviewed and analyzed by a senior gastrointestinal pathologist blinded to patient outcomes. Disease stage was determined using the TNM classification of the* American Joint Committee on Cancer 7th edition* [13]. The pathologic factors analyzed included lymphovascular invasion, invasive tumor pattern, grade of differentiation, mucin production, and intratumoral lymphocyte infiltration.

### 2.2. MicroRNA Microarray

Total RNA was extracted from the tumor tissues and normal tissues from ten gastric cancer patients (including 5 node-negative and 5 node-positive patients) and was prepared using mirVana miRNA Isolation kit (Invitrogen), according to the manufacturer's instruction. RNA concentration and purity were assessed by spectrophotometric analysis. The quality of small RNAs in each sample was determined using the 2100 Bioanalyzer assay (Agilent Technologies, Santa Clara, CA, USA). MiRNA microarray experiments were carried out by using the Agilent Human miRNA Microarray Kit version 3 (Based on Sanger miRbase release 12.0) with probe sets for 470 human miRNAs (miRBase release 9.1). For each sample, 100 ng total RNA was hybridized with the miRNA array and further processed according to Agilent's miRNA Microarray System protocol. The arrays were scanned with an Agilent Technology G2565B scanner (Agilent Technologies). The scanned images were gridded and analyzed by using Agilent Feature Extraction Software version 10.1.

### 2.3. Microarray Data Analysis

MiRNA-chip raw data were normalized separately using the GeneSpring software version 7.2 (Agilent). Both on chip and on gene median methods were used to normalize gene expression data. Microarray data were then joined into one GeneSpring genome and samples were assigned to one of two groups. The comparative analysis between node-positive and node-negative gastric cancer was carried out using Welch's *t*-test and the Benjamini & Hochberg or Bonferroni (for a more stringent analysis) False Discovery Rate correction. Cluster analysis was performed using the Pearson correlation as a measure of similarity. Predictions were made using both Support Vector Machine algorithm and PAM software. The Gene Ontology analysis of the gene lists of interest was generated by using the web delivered tools of Ingenuity Pathway Analysis. Data have been submitted to Array Express (Accession number EMEXP-326).

### 2.4. Real-Time PCR of Mature miRNAs

RT primers were designed for the mature miRNAs, which were significantly different in 5 node-positive and 5 node-negative gastric cancer patients' tissues by microRNA microarray hybridization. Because the amount of the remnant samples after first screening by miRNA microarray was not enough for the second screening by RT-PCR, we use another 46 samples for the screening by RT-PCR, including 23 node-positive and 23 node-negative patients. cDNAs were obtained from a stem-loop RT reaction using miRNA-specific stem-loop RT primers and SuperScript III Reverse Transcriptase, according to the manufacturer's manual (Invitrogen; Carlsbad, CA, USA). The reaction was performed using the following incubation conditions: 30 min at 16°C, followed by 50 cycles at 20°C/30 sec, 42°C/30 sec, and 50°C/1 sec. The enzyme was subsequently inactivated by incubation at 85°C for 5 min. The cDNA was used at a dilution of 1 : 20 in water in subsequent real-time PCR reactions. Real-time PCR reactions were performed using a specific forward primer and a universal reverse primer and were carried out by incubation at 94°C for 10 min, followed by 40 cycles of 94°C/15 sec and 60°C/32 sec. Gene expression was detected using a SYBR Green I assay (Applied Biosystems, Foster City, CA, USA) and the expression levels of miRNAs were normalized to that of U6 (DCt 1/4 target microRNA Ct-U6Ct). Expression levels of selected miRNAs in gastric cancer tissues were expressed as fold-change over adjacent normal tissues using the DDCt calculation (DDCt 1/4 DCt, tumor-DCt, adjacent normal) [14].

### 2.5. Statistical Analysis

All results in the text and tables are given as mean values ± standard deviation.

Categorical variables were analyzed using a Chi-square test with Yates' correction. Comparisons of quantitative variables between groups used Student's *t*-test. *P* < 0.05 for a 2-tailed test was considered significant. Statistical analysis used Statistical Package for the Social Sciences for Windows version 16.0 (SPSS, Inc., Chicago, IL, USA).

## 3. Results

MiRNA microarray analyses were performed for 10 patients initially, including 5 patients with LNM and 5 patients without LNM. The results of miRNA microarray showed ten miRNAs which were expressed significantly different between patients with or without LNM, including miR-451, miR-497, miR-1207-5p, miR-188-5p, miR-30a-5p, let-7e, let-7g, let-7f, miR-96, and let-7a.

### 3.1. The Correlation between miRNA and Lymph Node Metastasis

The ten miRNAs which were expressed significantly different between node positive and node negative patients in microarray analysis were selected for further analysis. A total of 46 patients were enrolled in this study, including 23 patients with LNM and 23 patients without LNM. As shown in Table 1, patients with upregulated miR-1207-5p were more likely to have no LNM than those with unchanged or downregulated miR-1207-5p (88.2% versus 25.9% versus 50%, *P* < 0.001). There was no significant correlation between lymph node metastasis and expression of other miRNAs.

### 3.2. The Correlation between miR-1207-5p and Pathological Characteristics

We further analyzed the correlation between the expression of miR-1207-5p and clinicopathological characteristics. As shown in Table 2, patients with upregulated miR-1207-5p had less scirrhous stromal reaction type, less lymphovascular invasion, and earlier pathological T category (*P* = 0.001), N category (*P* = 0.015), and TNM stage (*P* = 0.004) compared with those with downregulated and unchanged miR-1207-5p.

### 3.3. Analysis of the Risk Factors of Lymph Node Metastasis

As shown in Table 3, univariate analysis showed that stromal reaction type, lymphovascular invasion, pathological T category and TNM stage, and expression of miR-1207-5p were associated with LNM. Multivariate analysis confirmed that stromal reaction type, lymphovascular invasion, pathological T category, TNM stage, and expression of miR-1207-5p were independent risk factors of LNM.

## 4. Discussion

Our novel findings showed that upregulation of miR-1207-5p was more likely to be associated with no LNM in gastric cancer compared with no change or downregulation of miR-1207-5p. Furthermore, miR-1207-5p was significantly upregulated in earlier stage gastric cancer compared with more advanced gastric cancer. Further in vivo and in vitro study of the role of miR-1207-5p on target genes and its association with carcinogenesis and lymph node metastasis in gastric cancer are important.

The correlations between Agilent miRNA microarray results and qPCR results are generally excellent [15]. In comparison with other miRNA microarrays, such as Illumina and Affymetrix, the best qPCR-microarray correlation was seen with the Agilent platform [16]. In our study, ten miRNAs significantly related to LNM were selected by miRNA microarray; however only one miRNA was selected by RT-PCR. The possible reason is that the ten specimens used in the miRNA microarray are different from the 46 patients used in the screening by RT-PCR. Furthermore, all ten miRNAs selected by miRNA microarray were different from the previous study [12]. We should be careful to explain this. In addition to the different specimens used in the RT-PCR, the carcinogenesis in gastric cancer is complicated, which might involve different mechanisms and pathways while patients have lymph node metastasis. As a result, various miRNAs might be involved in LNM and carcinogenesis in gastric cancer. Furthermore, racial and environmental factors might have impact on the miRNAs associated with LNM.

We should also take into consideration whether there is difference of the expression of miRNAs among normal samples because of the contamination of intestinal metaplasia or dysplasia. Although the normal samples in the present study were all taken from the gastric mucosa at least 5 cm away from the margin of the gastric cancer, we could not completely exclude the possibility of contamination. Moreover, it was reported that the miRNA expression profile of gastric antrum was significantly correlated with gastric cardia, suggesting the presence of an “organ signature” in addition to a tissue signature [17]. It is interesting whether the LNM-related miRNAs are also similar among different tumor locations of gastric cancer. Our future study will enroll more patients for comparing the differences of LNM-related miRNA profiling between gastric cancers with different locations.

Human telomerase reverse transcriptase (hTERT) was one of the predicted target genes of miR-1207-5p. hTERT mRNA was reported to be upregulated in gastric cancer tissue, followed by dysplasia, intestinal metaplasia, and normal gastric mucosa (85% versus 53% versus 35% versus 0%) [18]. miR-1207-5p might regulate gastric carcinogenesis by targeting hTERT [19]. Kang et al. [20] reported that circulating cell-free hTERT mRNA was significantly higher in gastric cancer than in chronic atrophic gastritis and healthy controls. Moreover, higher level of circulating cell-free hTERT mRNA was significantly correlated with more advanced TNM stages, more lymph nodes metastasis, and decreased disease-free survival. Detection of hTERT mRNA could even serve as a tumor marker for detection of gastric cancer or tumor recurrence [21]. Furthermore, investigation of miR-1207-5p and its possible target gene, hTERT, in gastric cancer is required. Since higher level of circulating cell-free hTERT mRNA was associated with decreased disease-free survival [20], plasma miR-1207-5p might play an important role in tumor recurrence. Future study of the expression of plasma miR-1207-5p in gastric cancer and investigating whether plasma miR-1207-5p level correlates with tumor recurrence are required. Our results might shed light on the investigation of useful biomarkers for detection of gastric cancer recurrence in the future.

Since LNM in gastric cancer is associated with a poor prognosis, a useful biomarker might remind the surgeon of the possibility of LNM in high risk patients and the need for careful lymph node dissection for these patients during surgery. Future in vivo and in vitro investigation of miR-1207-5p in the mechanism of carcinogenesis and LNM of gastric cancer are required.

## 5. Conclusion

Our results showed that miR-1207-5p could serve as a useful biomarker in the prediction of LNM in gastric cancer.

## Figures and Tables

**Table 1:** The correlation between lymph node metastasis and miRNA in gastric cancer.

	Without LNM	With LNM	*P* value
	*n* = 23	*n* = 23	
	*n* (%)	*n* (%)	
miR-451			0.470
Unchanged	7 (41.2)	10 (58.8)	
Upregulated	11 (61.1)	7 (38.9)	
Downregulated	5 (45.5)	6 (54.5)	
miR-497			0.301
Unchanged	9 (60)	6 (40)	
Upregulated	13 (50)	13 (50)	
Downregulated	1 (20)	4 (80)	
miR-1207-5p			<0.001
Unchanged	7 (25.9)	20 (74.1)	
Upregulated	15 (88.2)	2 (11.8)	
Downregulated	1 (50)	1 (50)	
miR-188-5p			0.582
Unchanged	12 (46.2)	14 (53.8)	
Upregulated	7 (63.6)	4 (36.4)	
Downregulated	4 (44.4)	5 (55.6)	
miR-30a-5p			0.278
Unchanged	8 (42.1)	11 (57.9)	
Upregulated	14 (60.9)	9 (39.1)	
Downregulated	1 (25)	3 (75)	
Let-7e			0.414
Unchanged	10 (52.6)	9 (47.4)	
Upregulated	9 (50)	9 (50)	
Downregulated	1 (25)	4 (75)	
Let-7g			0.110
Unchanged	6 (31.6)	13 (68.4)	
Upregulated	15 (62.5)	9 (37.5)	
Downregulated	2 (66.7)	1 (33.3)	
Let-7f			0.497
Unchanged	15 (55.6)	12 (44.4)	
Upregulated	7 (46.7)	8 (53.3)	
Downregulated	1 (25)	3 (75)	
miR-96			0.068
Unchanged	12 (63.2)	7 (36.8)	
Upregulated	8 (57.1)	6 (42.9)	
Downregulated	3 (23.1)	10 (76.9)	
Let-7a			0.149
Unchanged	14 (60.9)	9 (39.1)	
Upregulated	8 (47.1)	9 (52.9)	
Downregulated	1 (16.7)	5 (83.3)	

LNM: lymph node metastasis.

**Table 2:** The clinicopathological characteristics between gastric cancer patients according to the expression of miR-1207-5p.

	Unchanged miR-1207-5p	Downregulated miR-1207-5p	Upregulated miR-1207-5p	*P* value
	*n* = 27	*n* = 2	*n* = 17	
	*n* (%)	*n* (%)	*n* (%)	
Age (y/o)				0.891
<65	9 (33.3)	1 (50)	6 (35.3)	
≧65	18 (66.7)	1 (50)	11 (64.7)	
Gender				0.890
M/F	16/11	1/1	11/6	
Tumor size (cm)				0.986
<5	14 (51.9)	1 (50)	10 (58.8)	
≧5	13 (48.1)	1 (50)	7 (41.2)	
Tumor location				0.421
Upper third stomach	4 (14.8)	0	4 (23.5)	
Middle third stomach	10 (37)	2 (100)	7 (41.2)	
Lower third stomach	13 (48.1)	0	6 (35.3)	
Lauren's classification				0.061
Intestinal type	14 (51.9)	0	13 (76.5)	
Diffuse type	13 (48.1)	2 (100)	4 (23.5)	
Cell differentiation				0.100
Poor	14 (51.9)	2 (100)	5 (29.4)	
Moderate/well	13 (48.1)	0	12 (70.6)	
Stromal reaction type				0.013
Medullary	1 (3.7)	1 (50)	5 (29.4)	
Intermediate	21 (77.8)	0	12 (70.6)	
Scirrhous	5 (18.5)	1 (50)	0	
Lymphovascular invasion				0.006
Absence	9 (33.3)	1 (50)	13 (76.5)	
Presence	18 (66.7)	1 (50)	4 (23.5)	
Pathological T categories				0.005
T1	1 (3.7)	1 (50)	10 (58.8)	
T2	9 (33.3)	0	3 (17.6)	
T3	14 (51.9)	1 (50)	4 (23.5)	
T4	3 (11.1)	0	0	
Pathological N categories				0.005
N0	7 (25.9)	1 (50)	14 (82.4)	
N1	7 (25.9)	0	0	
N2	6 (22.3)	0	2 (11.8)	
N3	7 (25.9)	1 (50)	1 (5.9)	
Pathological TNM stage				0.002
I	6 (22.2)	1 (50)	12 (70.6)	
II	10 (37)	0	4 (23.5)	
III	6 (22.2)	0	1 (15.2)	
IV	5 (18.6)	1 (50)	0	

**Table 3:** Univariate and multivariate analysis of risk factors of lymph node metastasis in gastric cancer.

	Without LNM	With LNM	*P* value	
	*n* = 23 *n* (%)	*n* = 23 *n* (%)	Univariate analysis	Multivariate analysis
Age (y/o)			0.758	
<65	9 (39.1)	7 (30.4)		
≧65	14 (60.9)	16 (69.6)		
Gender			0.130	
M/F	17/6	11/12		
Tumor size (cm)			0.236	
<5	15 (65.2)	10 (43.5)		
≧5	8 (34.8)	13 (56.5)		
Tumor location			0.767	
Upper third stomach	5 (21.7)	3 (13.6)		
Middle third stomach	9 (39.1)	10 (45.5)		
Lower third stomach	9 (39.1)	9 (40.9)		
Lauren's classification			0.071	
Intestinal type	17 (52.6)	10 (43.5)		
Diffuse type	6 (47.4)	13 (56.5)		
Cell differentiation			0.236	
Poor	8 (34.8)	13 (45.5)		
Moderate/well	15 (65.2)	10 (56.6)		
Stromal reaction type			<0.001	<0.001
Medullary	7 (30.4)	0		
Intermediate	16 (69.6)	17 (73.9)		
Scirrhous	0	6 (26.1)		
Lymphovascular invasion			<0.001	<0.001
Absence	18 (78.3)	5 (21.7)		
Presence	5 (21.7)	18 (78.3)		
Pathological T categories			<0.001	<0.001
T1	11 (47.8)	1 (4.3)		
T2	8 (34.8)	4 (17.4)		
T3	4 (17.4)	15 (65.2)		
T4	0	3 (13)		
Pathological TNM stage			<0.001	<0.001
I	19 (82.6)	0		
II	4 (17.4)	10 (43.5)		
III	0	7 (30.4)		
IV	0	6 (26.1)		
Expression of miR-1207-5p			<0.001	<0.001
No change	7 (30.4)	20 (87)		
Downregulation	1 (4.3)	1 (4.3)		
Upregulation	15 (65.2)	2 (8.7)		

LNM: lymph node metastasis.
